# Specific gut microbiome and metabolome changes in patients with continuous ambulatory peritoneal dialysis and comparison between patients with different dialysis vintages

**DOI:** 10.3389/fmed.2023.1302352

**Published:** 2024-01-05

**Authors:** Jiaqi Li, Haitao Xing, Wei Lin, Hangxing Yu, Bo Yang, Chen Jiang, Jin Zhang, Ruoxi Wu, Fengmei Ding, Ming Pei, Hongtao Yang

**Affiliations:** ^1^Department of Nephrology, First Teaching Hospital of Tianjin University of Traditional Chinese Medicine, Tianjin, China; ^2^Department of Nephrology, Xiamen Hospital of Traditional Chinese Medicine, Xiamen, China; ^3^Chongqing Hospital of Traditional Chinese Medicine, Chongqing, China

**Keywords:** continuous ambulatory peritoneal dialysis, dialysis vintage, gut microbiota, metabolite, uremic toxins, SCFAs

## Abstract

**Background:**

In recent years, the role of gut microbiota and derived metabolites in renal disease has attracted more attention. It has been established that the gut microbiota is a potential target for medical interventions in renal disease including chronic kidney disease (CKD), acute kidney injury (AKI) and renal calculus. Emerging evidence has related dialysis treatment to the microbial composition and function of the intestines, and there are many reports related to HD, but few studies have been related to PD. Previous studies have found that PD patients have intestinal flora disturbances, so we speculate that intestinal flora and its metabolites may be the regulatory factors in long-term therapy of PD. And as far as we know, there have been no studies characterized the gut microbiota in PD patients of different dialysis vintages.

**Methods:**

It is a cross-sectional study based on clinical data and biological samples of 72 patients with CAPD, 13 patients with ESRD and 13 healthy volunteers. The intestinal microecological characteristics of CAPD patients were comprehensively evaluated by combining the intestinal microflora structure, enterotoxin and receptor (serum LPS and LBP), intestinal barrier function index (serum D-Lactate), intestinal uremic toxin (serum IS, PCS, TMAO), fecal SCFAs and other multi-dimensional and multi-omics studies. Furthermore, the changes of intestinal microecology in CAPD patients of different dialysis vintages (≥ 3 and < 12 months, ≥ 12 and < 24 months, ≥ 24 and < 60 months, ≥ 60 months) were further explored, and the correlations between intestinal microecology indicators and some clinical indicators were analyzed. Fecal and serum samples were collected from PD patients (PD group, *n* = 72), ESRD patients (ESRD group, *n* = 13) and healthy volunteers (Normal group, *n* = 13). Fecal samples were subjected to microbiome (16S rDNA) and SCFA (GC-MS) analyses. Serum samples were subjected to LPS, LBP, D-lactate, IS, PCS, and TMAO (ELISA) analyses.

**Results:**

The diversity and richness of intestinal flora in CAPD patients were lower than those in healthy people and ESRD patients, and the microflora structure was different. Anaerobes of Blautia and facultative anaerobes and aerobic bacteria with Bacilli and Lactobacillales those in Firmicutes are the main intestinal flora in CAPD patients. The abundance of Bacteroidaceae, Bacteroides, Faecalibacterium and other dominant bacteria in the intestinal tract of CAPD patients decreased. Proteobacteria, Enterobacteriaceae and Escherichia-Shigella increased their colonization (LDA > 4). In CAPD patients of different dialysis vintages, there was no significant change in the diversity and richness of microflora, and the microflora structure of PDC group was significantly different from that of PDD, which the abnormal expansion of enterobacter group was more prominent in PDC and the abundance of Bacteroides group was relatively higher in PDD. Intestinal barrier damage, intestinal uremic toxin accumulation and short-chain fatty acid reduction were observed in CAPD patients, such as the serum level of D-Lactate, PCS and TMAO were significantly higher than that in the Normal group (*P* < 0.05),and the fecal levels of BA and CA were significantly lower (*P* < 0.05). The intestinal microecological disorder of PDC group, while that of PDD group showed a better trend. Such as the PDC group had a significantly higher serum level of LPS, D-Lactate and TMAO (*P* < 0.01), and significantly lower serum level of LBP (*P* < 0.01), and lower fecal levels of AA and BA (*P* > 0.05) than the PDD group.

**Conclusion:**

The intestinal microecology and metabolic system of CAPD patients had changes compared with healthy people and ESRD non-dialysis patients, and there were differences in CAPD patients with different dialysis vintages. PD patients on dialysis for more than 60 months showed a better trend in the intestinal microecology than patients with 24∼36 months, which suggested that the intestinal microecology of PD patients had a certain ability of self-regulation and remodeling under the management of standardized system and it is necessary to strengthen the monitoring of the intestinal status and the occurrence of related complications in PD patients on dialysis of 24∼36 months of dialysis vintage. It is initially considered that the mechanism of intestinal microecology is a potential target for intervention in the diagnosis and treatment of CAPD and incorporating intestinal microecosystem monitoring into the long-term management of CAPD patients is a new strategy.

## 1 Introduction

Peritoneal dialysis (PD) is an effective renal replacement therapy for the long-term survival of patients with renal failure. Compared with hemodialysis (HD), it has the advantages of simple operation, relatively low cost and better protection of residual renal function. Especially during the coronavirus disease 2019 (COVID-19) epidemic, PD has demonstrated its unique advantages in public emergencies as the leading form of home-based treatment (1, 2). Indeed, patient outcomes with PD are comparable to or better than those with HD. PD has been used in many countries around the world, and approximately 11% of people with kidney failure worldwide are treated with PD. Although large inter- and intraregional disparities exist in PD availability, accessibility, affordability, delivery, and reporting of quality outcome measures around the world, use of this therapy is increasing in some countries, including China, the USA and Thailand. (3, 4) Therefore, extending the duration of PD application and improving efficiency are still the main topics in the field of nephrology.

In recent years, the role of gut microbiota and derived metabolites in renal disease has attracted more attention. It has been established that the gut microbiota is a potential target for medical interventions in renal disease including chronic kidney disease (CKD), acute kidney injury (AKI) and renal calculus (5, 6). Emerging evidence has related dialysis treatment to the microbial composition and function of the intestines, and there are many reports related to HD (7–10), but few studies have been related to PD. In the treatment of PD, many factors will affect the gut environment of PD patients. Patients with PD have prolonged intestinal contact with peritoneal dialysis fluid and are in the unique abdominal environment of high pressure and high sugar levels. Most PD patients have lost all or only partial residual renal function, and the toxins that cannot be excreted by the kidney will be passively metabolized through the intestine. The combination of drugs such as calcium and phosphorus binders in PD treatment is also a factor. Although some studies have preliminarily confirmed that the composition, abundance and functional evolution of the gut microbiota in PD patients have changed (11, 12), but no studies have characterized the gut microbiota in PD patients of different dialysis vintages. The different roles of intestinal microecological mechanisms at different stages of PD long term treatment may be targets for intervention.

In addition, CKD is characterized by reduced clearance rate and increased serum accumulation of metabolic waste, namely uremic solute retention, of which gut-derived metabolites are important sources. Intestinal flora is directly involved in various metabolic and synthetic processes of human body, and most of the end products are toxic. These metabolites play a variety of pathophysiological roles both in the intestine and extra-intestine, and their levels and proportions are affected by intestinal flora. Gut derived uremic toxins such as indoxyl sulfate (IS) (13, 14), p-cresol sulfate (PCS) (14–16) and trimethylamine oxide (TMAO) (17–19) have been shown to accumulate excessively in the intestine and blood of patients with chronic kidney disease (CKD) and end-stage renal disease (ESRD) and to play toxic effects. Short-chain fatty acids (SCFAs), which are major metabolites in the gut have also been proven to affect the progression of kidney disease (20–22). We speculate that changes in gut-derived metabolites caused by alterations of the gut microbiome may also play an important role in the long-term treatment of PD. Together, comprehensive microbiology and metabolomic analyses may thus be useful for a better application of PD therapy through associated alterations in the gut ecosystem.

## 2 Materials and methods

### 2.1 Source of population

The clinical study was approved by the Institutional Review Board of First Teaching Hospital of Tianjin University of Traditional Chinese Medicine (approval No. TYLL2021[K] 009). Informed consent was obtained before the study commenced in accordance with the Declaration of Helsinki.

The study subjects were CAPD patients regularly managed by the Peritoneal Dialysis Center of the Department of Nephrology, the First Affiliated Hospital of Tianjin University of Chinese Medicine, from June 2021 to December 2021. We adopted a stratified sampling method and divided them into 4 groups according to the dialysis vintage, including 18 patients with dialysis vintages of 3∼12 months (PDA group), 18 patients with dialysis vintages of 12∼24 months (PDB group), 18 patients with dialysis vintages of 24∼36 months (PDC group), and 18 patients with dialysis vintages of 60 months and above (PDD group), for a total of 72 patients. Another 13 ESRD patients (ESRD group) and 13 healthy individuals (Normal group) were selected as the control group. The diagnosis criteria refer to the 2012 KDIGO (23) for the new definition of CKD and the CGA staging system, CKD patients have abnormal kidney structure or function and affect health (one of the criteria can be met: albuminuria: AER > 30 mg/24 h or ACR > 30 mg/g; abnormal urinary sediment; abnormal electrolyte or other abnormalities due to renal tubule lesions; abnormal histopathology; structural abnormalities found on imaging; history of renal transplantation >3 months or more), eGFR < 15 ml/min/1.73 m^2^ or dialysis (with or without renal damage) can be diagnosed as ESRD. In the general CAPD treatment mode, the dialysate was exchanged 3 to 5 times a day, each time 1.5 to 2 L of dialysate was used, and the dialysate was retained in the abdominal cavity for 4–6 h during the day and 10–12 h at night. The inclusion criteria of the PD group were as follows: (I) patients aged 18–75 years old and (II) patients with clearly diagnosed ESRD and regular CAPD treatment for 90 days or more. The inclusion criteria of the ESRD group were as follows: (I) patients aged 18–75 years old and (II) patients with clearly diagnosed ESRD who were not undergoing PD, HD, kidney transplantation, or any other kidney replacement therapy. The inclusion criteria of the Normal group were as follows: (I) people aged 18–75 years old and (II) people who had completed their physical examination and were eligible for the improved SENIEUR protocol (24). To exclude the interference of the living environment, 13 healthy people were family members of PD patients, twelve of whom were spouses and one was a live-in child. Thirteen ESRD patients were also treated in the outpatient clinic or ward of our hospital during the same period. Excluding the interference of diabetes on the intestinal flora, all 98 participants had normal blood glucose. All 98 participants had no history of infection and no history of antibiotic use in the past 2 weeks. In addition, a washout period of 2 weeks was set up before specimen collection to ensure that all participants did not consume probiotics, prebiotics, or synbiotics for the last 2 weeks, and all participants received diet interviews and had regular 3-meal dietary patterns. Dietary guidance was given during washout according to the KDOQI clinical practice guideline for nutrition in CKD updated in 2020 (25). The specific method was to record diet for 3 days per week (including 2 times on weekdays and 1 time on weekends). In the washout period, 24-h diet review method was used to record the diet 3 times a week (2 times on weekdays, 1 time on weekends), and the sample was collected after 2 weeks of continuous recording.

### 2.2 Human specimens

Fresh fecal samples were collected from patients with sterile fecal collection tubes and stored at −80°C until DNA extraction. Blood samples were collected on an empty stomach from all patients in the morning, and 5 ml of fresh venous blood was drawn from the cubital vein. Blood samples were centrifuged at 3500 × rpm for 10 min at room temperature, and then serum was harvested and stored at −80°C until use.

### 2.3 Biochemical measurements

Demographic and clinical data of PD patients were collected from medical records, including sex, age, and body mass index (BMI). Clinical parameters included fasting blood sugar (FBG),hemoglobin (HGB), platelet (PLT), white blood cell (WBC), neutrocyte, lymphocyte, monocytes, albumin (ALB), triglyceride (TG), total cholesterol (TC),blood urea nitrogen (BUN), creatinine (CR), serum uric acid (UA), serum phosphate, serum potassium, Peritoneal Kt/V (PD Kt/V), residual renal Kt/V, Peritoneal CCr, residual renal CCr, total CCr standardization and normalized protein catabolic rate (nPCR). Serum levels of lipopolysaccharide (LPS), Lipoprotein binding protein (LBP) and D-Lactate were measured by ELISA in humans.

### 2.4 Measurements of gut microbiota

Total genomic DNA from stool samples was extracted using the cetyltrimethylammonium bromide or sodium dodecyl sulfate (CTAB/SDS) method. DNA concentration and purity were monitored on 1% agarose gels. According to the concentration, DNA was diluted to 1 ng/μL using sterile water. The V4 region of the 16S rRNA gene was amplified using specific primers (515F: GTGCCAGCMGCCGCGGTAA and 806R: GGACTACHVGGGTWTCTAAT) with barcodes. All PCR mixtures contained 15 μL of Phusion^®^ High-Fidelity PCR Master Mix (New England Biolabs), 0.2 μM of each primer and 10 ng target DNA, and cycling conditions consisted of a first denaturation step at 98°C for 1 min, followed by 30 cycles at 98°C (10 s), 50°C (30 s) and 72°C (30 s) and a final 5 min extension at 72°C. An equal volume of 1X loading buffer (containing SYBR Green) was mixed with the PCR products, and electrophoresis was performed on a 2% agarose gel for DNA detection. The PCR products were mixed in equal proportions, and then a Qiagen Gel Extraction Kit (Qiagen, Germany) was used to purify the mixed PCR products. Following the manufacturer’s recommendations, sequencing libraries were generated with the NEBNext^®^ UltraTM IIDNA Library Prep Kit (Cat No. E7645). The library quality was evaluated on a Qubit@ 2.0 Fluorometer (Thermo Scientific) and Agilent Bioanalyzer 2100 system. Finally, the library was sequenced on an Illumina NovaSeq 6000 platform, and 250 bp paired-end reads were generated. The paired-end reads were assigned to samples based on their unique barcodes before cutting off the barcodes and primers. Paired-end reads were merged using FLASH software (V 1.2.11) (26). Quality filtering of the raw tags was performed using fastp (Version 0.20.0) software to obtain high-quality clean tags. The clean tags were compared with the reference database (Silva database) using Vsearch (V 2.15.0) to detect the chimera sequences, and then the chimera sequences were removed to obtain the effective tags (27). For the effective tags obtained previously, denoising was performed with DADA2 in QIIME2 software (V 2-202006) to obtain initial ASVs (Amplicon Sequence Variants), and then ASVs with abundance less than 5 were filtered out (28). The absolute abundance of ASVs was normalized using a standard sequence number corresponding to the sample with the fewest sequences. To analyze the diversity, richness and uniformity of the communities in the sample, alpha diversity was calculated from 6 indices in QIIME2, including Observed-otus, Chao1, Shannon, Simpson, Good’s coverage and Pielou-e. To evaluate the complexity of the community composition and compare the differences between groups, beta diversity was calculated based on weighted UniFrac distances in QIIME2. Principal coordinate analysis (PCoA) was performed to obtain principal coordinates and visualize differences in samples in complex multidimensional data using R software (V 2.15.3). To study the significance of the differences in community structure between groups, the ANOSIM functions in QIIME2 software were used for analysis. LEfSe software (V 1.0) was used to perform LEfSe analysis (LDA score threshold: 4) to determine the biomarkers. Subsequently, the predicted functional composition profiles of 16S rRNA sequences were collapsed into Kyoto Encyclopedia of Genes and Genomes (KEGG) pathways using Tax4Fun (29).

### 2.5 Measurements of metabolites

Serum levels of IS, PCS, and TMAO were measured by ELISA in humans. Agilent 7890B gas chromatograph coupled to a 7000D mass spectrometer with a DB-FFAP column (30 m length × 0.25 mm i.d. × 0.25 μm film thickness, J&W Scientific, USA) was employed for GC-MS/MS analysis of SCFAs. Helium was used as the carrier gas at a flow rate of 1.2 mL/min. Injection was performed in split mode, and the injection volume was 2 μL. The oven temperature was held at 90°C for 1 min, raised to 100°C at a rate of 25°C/min, then raised to 150°C at a rate of 20°C/min, held for 0.6 min, raised to 200°C at a rate of 25°C/min, held for 0.5 min, after running for 3 min. All samples were analyzed in multiple reaction monitoring mode. The injector inlet and transfer line temperatures were 200 and 230°C, respectively (30–32).

### 2.6 Statistical analysis

All statistical analyses were performed using SPSS Statistics software (version 26.0; IBM Corp). SPSS26.0 was used for statistical analysis of experimental data. The data were described in terms of frequency, mean ± SD. The chi-square test, Fisher’s exact probability test and rank sum test were used to compare the count data between groups. Tests of normality and homogeneity of variance were performed before comparison of data in each group. For data conforming to a normal distribution, one-way ANOVA was used for comparisons among multiple groups, Tukey’s test was used for homogeneity of variances, and Dunnett’s T3 was used for heterogeneity of variances. A non-parametric test was used for skewed data, and the Kruskal-Wallis *H*-test was used for comparisons among multiple groups. Correlation analysis was performed by Spearman or Pearson’s tests. The chart was constructed by R software (V 2.15.3) and GraphPad Prism 9. Statistical significance was established at *p*-values < 0.05.

## 3 Results

This study was based on a cross-sectional study of clinical data and biological samples from 72 CAPD patients, 13 ESRD patients and 13 healthy volunteers. Intestinal microecological characteristics of CAPD patients were comprehensively evaluated by combining the results of multi-dimensional and multi-omics studies on intestinal flora structure, enterogenic endotoxin and receptor (serum LPS and LBP), intestinal barrier function index (serum D-Lactate), enterogenic uremic toxin (serum IS, PCS, TMAO), fecal SCFAs, etc. Furthermore, the changes of intestinal microecology in CAPD patients of different dialysis ages (≥ 3 and < 12 months, ≥ 12 and < 24 months, ≥ 24 and < 60 months, ≥ 60 months) were further explored, and the correlation between intestinal microecology indicators and some clinical indicators was analyzed.

### 3.1 Baseline characteristics of participants

A total of 98 participants were enrolled. There were 63 male participants and 35 female participants in this study. The six groups did not differ significantly in age or BMI. The four groups of PD patients differed significantly in PD vintage, with the shortest being 6 months and the longest being 107 months. All 98 participants had normal FBG levels. Except for the absolute value of lymphocytes and serum iPTH (*P* < 0.05), no significant differences in other biochemical indicators between the PD groups of different dialysis vintages were found. The baseline characteristics of the study population are described in Table 1 and Supplementary Table 1.

**TABLE 1:** Clinical characteristics of the study population.

Variables	Normal	ESRD	PD				*p-*value
			**PDA**	**PDB**	**PDC**	**PDD**	
No.(male/female)	13 (8/5)	13 (9/4)	18 (10/8)	18 (14/4)	18 (15/3)	18 (7/11)	
Age	47.85 ± 8.09	54.62 ± 5.75	46.72 ± 9.38	47.22 ± 10.64	49.19 ± 9.19	53.83 ± 12.43	0.088
BMI (kg/m2)	23.63 ± 2.39	23.78 ± 1.57	25.14 ± 3.87	26.26 ± 9.81	24.97 ± 3.18	21.44 ± 5.53	0.145
FBG (mmol/L)	4.71 ± 0.50	5.19 ± 0.44	5.23 ± 0.60	5.27 ± 0.45	4.97 ± 0.66	5.66 ± 0.77	
PD vintage (months)			8.39 ± 1.79	19.11 ± 3.76	32.83 ± 2.64	71.72 ± 13.68	

Values are expressed as absolute number or mean ± SD.

### 3.2 Gut microbiome characteristics of CAPD patients and subgroup analysis of different dialysis vintages

16S rRNA gene sequencing was used to evaluate the alterations in the gut microbiome triggered by PD treatment and exposure to different PD vintages. Community diversity and richness were calculated by alpha diversity metrics. The richness index included the Observation-Otus and Chao1 indices, the diversity index included the Shannon and Simpson indices, and the Pielou-e index was used to evaluate species evenness. In addition, the Goods-coverage index was used to assess sequencing depth. The Shannon index and Pielou-e index indicated that there were significant differences in the diversity and evenness of the gut microbiome in the Normal, ESRD and PD groups (*P* < 0.05), and the evenness of the gut microbiome in the PD group was significantly lower than that in the Normal (*P* = 0.042) and ESRD (*P* = 0.021) groups by the Pielou-e index. In the analysis of PD subgroups, the Observed-otus, Chao1, Shannon, Simpson and Pielou-e indexes showed no significant difference among the PDA, PDB, PDC and PDD groups (*P* > 0.05). The results showed that there were no significant differences in the richness, diversity and evenness of the gut microbiome in PD patients of different dialysis vintages. The Goods-coverage index of all samples was 1, indicating that the sequencing depth of the samples covered most bacteria in the fecal flora. The alpha indexes between the Normal, ESRD, and PD groups or between the four PD subgroups are described in Tables 2, 3.

**TABLE 2:** Alpha index in the Normal, ESRD, and PD groups.

Variables	Alpha index					
	**Richness**		**Diversity**		**Evenness**	**Sequencing depth**
	**Chao1**	**Observed-otus**	**Shannon**	**Simpson**	**Pielou-e**	**Goods-coverage**
Normal	219.8 ± 30.01	219.5 ± 29.92	5.75 ± 0.503	0.95 ± 0.024	0.74 ± 0.058	1
ESRD	229.8 ± 39.94	229.6 ± 39.94	5.85 ± 0.449	0.95 ± 0.028	0.75 ± 0.048	1
PD	233.6 ± 55.40	232.8 ± 55.38	5.32 ± 0.841	0.91 ± 0.089	0.68 ± 0.093*/^#^	1
*F*-value	0.407	0.376	3.821	2.158	5.811	
*P*-value	0.667	0.688	0.025	0.121	0.004	

Simpson Index is Simpson’s Index of Diversity (1-D); Values are expressed as absolute number or mean ± SD; Compared with Normal group,

*P < 0.05; Compared with ESRD group,

^#^*P* < 0.05.

**TABLE 3:** Alpha index in the PDA, PDB, PDC, and PDD groups.

Variables	Alpha index					
	**Richness**		**Diversity**		**Evenness**	**Sequencing depth**
	**Chao1**	**Observed-otus**	**Shannon**	**Simpson**	**Pielou-e**	**Goods-coverage**
PDA	231.9 ± 69.77	230.9 ± 69.95	5.03 ± 1.010	0.89 ± 0.104	0.64 ± 0.105	1
PDB	247.9 ± 66.01	246.5 ± 66.34	5.41 ± 0.753	0.93 ± 0.043	0.68 ± 0.072	1
PDC	230.3 ± 41.03	230.1 ± 40.92	5.38 ± 0.776	0.91 ± 0.102	0.69 ± 0.092	1
PDD	224.3 ± 39.85	223.8 ± 39.27	5.45 ± 0.810	0.92 ± 0.094	0.70 ± 0.098	1
*F*-value	0.582	0.537	0.931	0.594	1.284	
*P*-value	0.629	0.658	0.431	0.621	0.287	

Simpson Index is Simpson’s Index of Diversity (1-D); Values are expressed as absolute number or mean ± SD.

**FIGURE 1:**
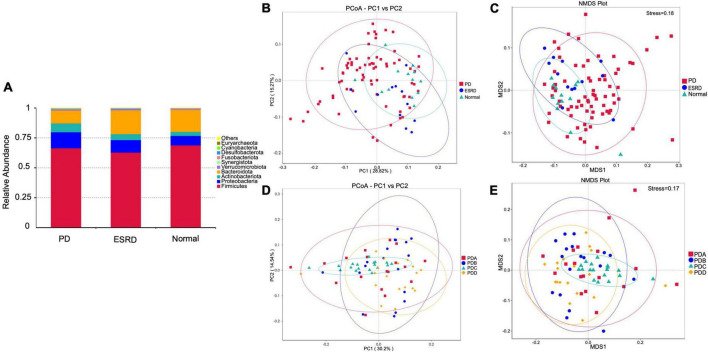
Gut microbiome structure analysis. **(A)** Composition and relative abundance of bacterial phyla in the Normal, ESRD and PD groups. **(B,D)** The PCoA plot is generated of the weighted UniFrac distance based on OTU counts and explains the largest variance between all samples. **(C,E)** Beta diversity was measured by NMDS analysis.

By comparison with the database Silva138, species annotation was carried out. The kingdom, phylum, class, order, family, genus and species were annotated according to the annotation results of ASVs and the feature table of each sample, and the absolute abundance before homogenization and the relative abundance after homogenization were obtained for each group at each taxonomic level. At the phylum level, Firmicutes was the predominant phylum in the gut microbiome in each of the three groups (Normal group, ESRD group, and PD group), followed by Bacteroidetes, Proteobacteria, and Actinobacteria, accounting for more than 95% (Figure 1A). Compared with the Normal and ESRD groups, the relative abundance of Proteobacteria in the PD group was higher (*P* > 0.05), and the relative abundance of Bacteroidetes was significantly lower (*P* < 0.05). At the genus level, compared with the Normal group, the relative abundance of Escherichia-Shigella increased in the ESRD and PD groups (*P* > 0.05), and the relative abundance of Faecalibacterium decreased significantly (*P* < 0.05). Compared with the Normal and ESRD groups, the relative abundance of Bacteroides in the PD group decreased significantly (*P* < 0.05). The PD group had a significantly higher relative abundance of Blautia than the Normal group (*P* > 0.05). The relative abundances of some bacteria at the phylum and genus levels among the three groups are shown in Table 4. In the subgroup analysis, the relative abundances of Proteobacteria and Escherichia-Shigella in the PDC group were significantly higher than those in the PDA, PDB, and PDD groups (*P* < 0.05). The relative abundances of Bacteroidetes, Bacteroides and Faecalibacterium in the PDD group were significantly higher than those in the PDA, PDB, and PDC groups (*P* < 0.05). The relative abundances of some bacteria at the phylum and genus levels among the four PD groups are shown in Table 5.

**TABLE 4:** The relative abundance of some bacteria in the normal, ESRD, and PD groups.

	Normal	ESRD	PD	*F*-value	*P*-value
**Phylum**					
Proteobacteria	0.079 ± 0.071	0.105 ± 0.114	0.134 ± 0.180	0.723	0.488
Actinobacteriota	0.034 ± 0.031	0.050 ± 0.033	0.074 ± 0.111	1.096	0.338
Bacteroidota	0.183 ± 0.064	0.200 ± 0.110	0.108 ± 0.089*/^##^	8.461	0.000
**Genus**					
*Escherichia-Shigella*	0.047 ± 0.068	0.090 ± 0.103	0.115 ± 0.176	1.079	0.344
*Faecalibacterium*	0.218 ± 0.074	0.118 ± 0.051**	0.133 ± 0.105**	4.793	0.010
*Streptococcus*	0.005 ± 0.004	0.011 ± 0.008	0.028 ± 0.055	1.670	0.194
*Bacteroides*	0.152 ± 0.056	0.154 ± 0.095	0.080 ± 0.076**/^##^	8.726	0.000
*Blautia*	0.044 ± 0.018	0.079 ± 0.057	0.099 ± 0.061***	5.278	0.007
*Ruminococcus_gnavus_group*	0.006 ± 0.005	0.011 ± 0.009	0.030 ± 0.045	3.054	0.052

Values are expressed as the absolute number or mean ± SD; Compared with the Normal group,

*P < 0.05,

**P < 0.01,

***P < 0.001; compared with the ESRD group,

^##^*P* < 0.01.

**TABLE 5:** The relative abundance of some bacteria of the PDA, PDB, PDC, and PDD groups.

	PDA	PDB	PDC	PDD	*H*-value	*P*-value
**Phylum**						
Proteobacteria	0.164 ± 0.233*	0.072 ± 0.111**	0.195 ± 0.164^#^	0.106 ± 0.180*	12.892	0.005
Actinobacteriota	0.117 ± 0.179	0.077 ± 0.094	0.037 ± 0.032	0.066 ± 0.079	3.447	0.328
Bacteroidota	0.100 ± 0.086^#^	0.119 ± 0.109^#^	0.053 ± 0.026^###^	0.161 ± 0.08***	15.903	0.001
**Genus**						
*Escherichia-Shigella*	0.138 ± 0.219*	0.054 ± 0.110***	0.173 ± 0.169^#^	0.097 ± 0.181*	14.209	0.003
*Faecalibacterium*	0.126 ± 0.120	0.158 ± 0.132	0.086 ± 0.046	0.163 ± 0.091	6.164	0.104
*Bacteroides*	0.079 ± 0.079^#^	0.080 ± 0.087^#^	0.036 ± 0.023^###^	0.123 ± 0.077***	15.396	0.002

Values are expressed as absolute number or mean ± SD; Compared with PDC group,

*P < 0.05,

**P < 0.01,

***P < 0.001; Compared with PDD group,

^#^*P* < 0.05,

^###^*P* < 0.001.

The PCoA plot showed that a distinct structure of the gut microbiome in the normal group, ESRD group and PD group was successfully separated, with 28.82 and 15.27% variation explained by the PC1 and PC2 principal components, respectively (Figure 1B). NMDS (non-metric multidimensional scaling) analysis also proved that there were certain differences between the three groups (Figure 1C). In subgroup analysis, the PCoA plot showed that a distinct structure of the gut microbiome in the PDA group, PDB group, PDC group, and PDD group was successfully separated, with 30.2 and 14.54% variation explained by the PC1 and PC2 principal components, respectively (Figure 1D). The NMDS analysis also proved that there were certain differences among the four groups (Figure 1E). The ANOSIM analysis showed that the bacterial community structure of the PDC group was significantly different from that of the PDA, PDB, and PDD groups (PDA vs. PDC: *R* = 0.146, *P* = 0.005; PDB vs. PDC: *R* = 0.183, *P* = 0.005; PDC vs. PDD: *R* = 0.362, *P* = 0.005).

**FIGURE 2:**
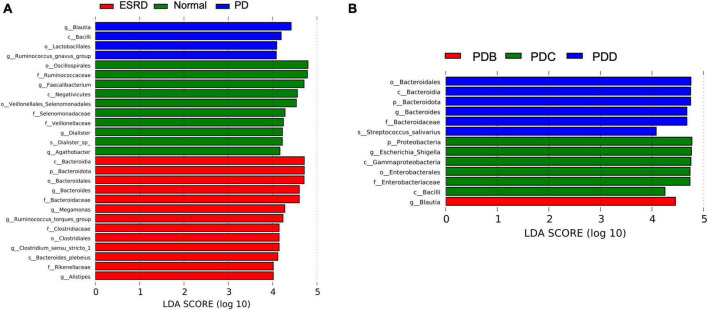
Differences in the gut microbiome between groups by LEfSe analysis. **(A,B)** The differences in abundance between the ESRD, normal and PD groups or the PDB, PDC, and PDD groups.

LEfSe analysis was used to compare the gut microbial community between groups (LDA score > 4). Oscillospirales, Ruminococcaceae, Faecalibacterium and Negativicutes were the predominant flora in the Normal group. Bacteroidia, Bacteroidota, Bacteroidales, Bacteroides and Bacteroidaceae were the predominant flora in the ESRD group. The Blautia, Bacilli, Lactobacillales and Ruminococcus gnavus groups were the predominant flora in the PD group (Figure 2A). In the subgroup analysis, LEfSe analysis showed that there were significant differences in species between the PDB, PDC, and PDD groups. The dominant microbiota in the PDB group was Blautia, and Bacteroidales, Bacteroidia, Bacteroidota, Bacteroides and Bacteroidaceae were the predominant flora in the PDD group, while the dominant microbiota in the PDC group were Proteobacteria, Escherichia-Shigella, Gammaproteobacteria, Enterobacterales, Enterobacteriaceae, and Bacilli (Figure 2B).

### 3.3 Functional prediction of the predominant taxa

**FIGURE 3:**
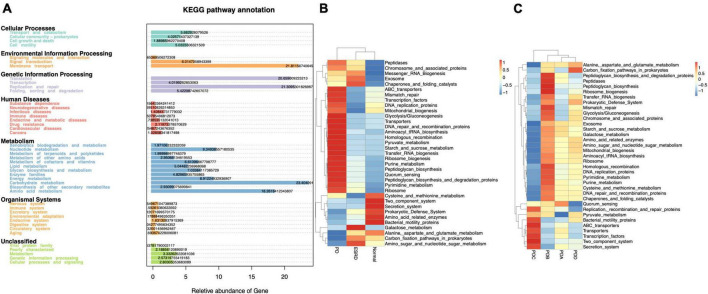
Functional annotation of the gut microbiome by Tax4Fun analysis. **(A)** Annotated diagram of KEGG pathway function. **(B,C)** Heatmap of the top 35 functional abundances between groups.

Tax4Fun is an open-source R package designed to estimate the functional capabilities of microbial communities identified in 16S rRNA gene sequencing, and it allowed functional annotation of the gut microbiome in the datasets of the Normal, ESRD, and PD groups. Tax4Fun function prediction connects the composition with the function of the microbiome. The results showed that the functional components of KEGG Level 1 were closely concentrated in metabolism. Carbohydrate metabolism and amino acid metabolism played a major role in the three groups (Figure 3A). Eight metabolic pathways, which were level 3 KEGG pathways, were differentially enriched between the three groups. The metabolic functions of the PD group were mainly pyruvate metabolism, starch and sucrose metabolism, pyrimidine metabolism and purine metabolism. Compared with the Normal and ESRD groups, the metabolic functions of alanine, aspartic acid and glutamate were less enriched (Figure 3B), and the PDC group had higher pyruvate metabolism enrichment abundance than the other three PD groups (Figure 3C).

### 3.4 Serum levels of LPS, LBP, and D-lactate in CAPD patients and subgroup analysis of different dialysis vintages

**FIGURE 4:**
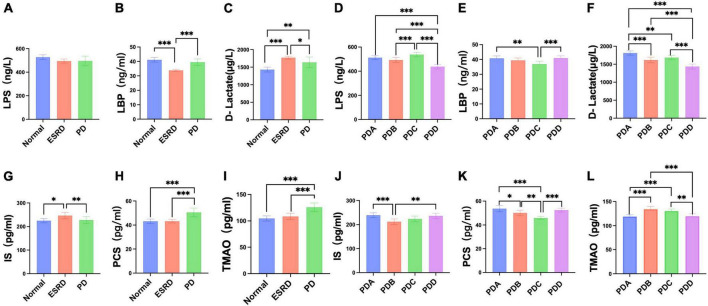
Differences in serum indicators (LPS, LBP, D-lactate, IS, PCS, TMAO) between groups. **(A–F)** Column bar graph illustrating the significant differences in content between the serum levels of LPS, LBP, and D-lactate, Tukey’s test, *P < 0.05, **P < 0.01, ***P < 0.001. **(G–L)** Column bar graph illustrating the significant difference in content between the serum levels of IS, PCS, and TMAO, Tukey’s test, *P < 0.05, **P < 0.01, ***P < 0.001.

Serum levels of LPS, LBP and D-lactate were assessed by ELISA. The serum levels of LPS, LBP and D-lactate between the Normal, ESRD and PD groups and between the four groups in the PD group are described in Supplementary Tables 2, 3. No differences were observed in the serum levels of LPS among the Normal, ESRD and PD groups (*P* > 0.05) (Figure 4A), while significant differences in serum levels of LBP and D-lactate were found (*P* < 0.0001) (Figures 4B, C). The serum levels of LBP in the Normal and PD groups were significantly higher than that in the ESRD group (*P* < 0.0001), while there was no significant difference between the Normal and PD groups (*P* > 0.05) (Figure 4B). The serum level of D-lactate in the PD group was significantly lower than that in the ESRD group (*P* = 0.049) but was still significantly higher than that in the Normal group (*P* = 0.002) (Figure 4C). In PD patients, the PDD group had a significantly lower serum level of LPS than the PDA, PDB, and PDC groups (*P* < 0.0001), and the PDC group exhibited a significantly higher serum level of LPS than the PDB group (*P* = 0.0005) (Figure 4D). The serum levels of LBP in the PDA (*P* = 0.0013) and PDD (*P* = 0.0008) groups were significantly higher than those in the PDC group (*P* < 0.01) (Figure 4E). The serum level of D-lactate in the PDD group was significantly lower than that in the PDA, PDB, and PDC groups (*P* ≤ 0.0001) (Figure 4F).

### 3.5 Serum levels of gut-derived uremic toxins in CAPD patients and subgroup analysis of different dialysis vintages

Serum levels of gut-derived uremic toxins were assessed by ELISA. The serum levels of gut-derived uremic toxins between the normal, ESRD and PD groups and between the four groups in the PD group are described in Supplementary Tables 4, 5. The ESRD group had a significantly higher serum level of IS than the normal (*P* = 0.0190) and PD (*P* = 0.0084) groups, but there were no differences between the normal and PD groups (*P* > 0.05) (Figure 4G). The serum levels of PCS and TMAO in the PD group were significantly higher than those in the normal and ESRD groups (*P* < 0.0001) (Figures 4H, I). In PD patients, serum levels of IS in the PDA and PDD groups were higher than those in the PDC group (*P* > 0.05) and significantly higher than those in the PDB (*P* = 0.0007, *P* = 0.0020) group (Figure 4J). The serum levels of PCS in the PDA, PDB, and PDD groups were all significantly higher than those in the PDC group (*P* ≤ 0.0001, *P* = 0.0031, *P* ≤ 0.0001) (Figure 4K). Serum levels of TMAO in the PDB and PDC groups were all significantly higher than those in the PDA (*P* ≤ 0.0001) and PDD (*P* = 0.0007, *P* = 0.0017) groups (Figure 4L).

### 3.6 Serum levels of fecal SCFAs of CAPD patients and subgroup analysis of different dialysis vintages

**FIGURE 5:**
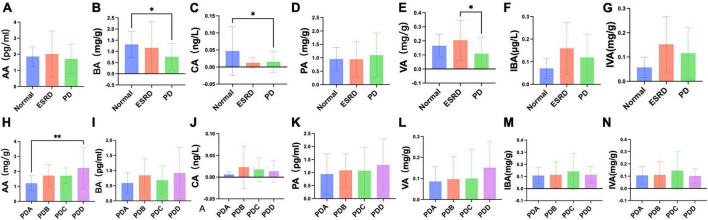
Differences in fecal levels of SCFAs between groups. **(A–N)** Column bar graph illustrating the significant difference in content between the fecal levels of SCFAs, Tukey’s test, *P < 0.05, **P < 0.01.

Fecal levels of SCFAs were assessed by gas chromatography-mass spectrometry (GC-MS). The fecal levels of SCFAs between the Normal, ESRD and PD groups and between the four groups in the PD group are described in Supplementary Tables 6, 7. There were no significant differences in the fecal levels of acetic acid (AA) and propionic acid (PA) between the Normal, ESRD and PD groups (*P* > 0.05) (Figures 5A, E), and the differences in the fecal levels of butyric acid (BA), caproic acid (CA) and valeric acid (VA) between the three groups were significant (*P* < 0.05) (Figures 5B–D). The fecal levels of BA and CA in the PD group were significantly lower than those in the Normal group (*P* = 0.024, *P* = 0.016) (Figures 5B, C), and the fecal level of VA in the PD group was significantly lower than that in the ESRD group (*P* = 0.018) (Figure 5D). The fecal levels of isobutyric acid (IBA) and isovaleric acid (IVA) in the PD group were higher than those in the Normal group but lower than those in the ESRD group, with no statistical significance (*P* > 0.05) (Figures 5F, G). In CAPD patients, the fecal levels of AA and BA in the PDB and PDD groups were higher than those in the PDA and PDC groups (Figures 5H, I), and the fecal level of AA in the PDD group was significantly higher than that in the PDA group (*P* = 0.003) (Figure 5H). There were no significant differences in the fecal levels of CA, VA, and PA (*P* > 0.05) (Figures 5J–L). The fecal levels of IBA and IVA in the PDC group were higher than those in the PDA, PDB, and PDC groups, and there was no statistical significance (*P* > 0.05) (Figures 5M, N).

### 3.7 Correlation analysis between indicators of the CAPD population

**FIGURE 6:**
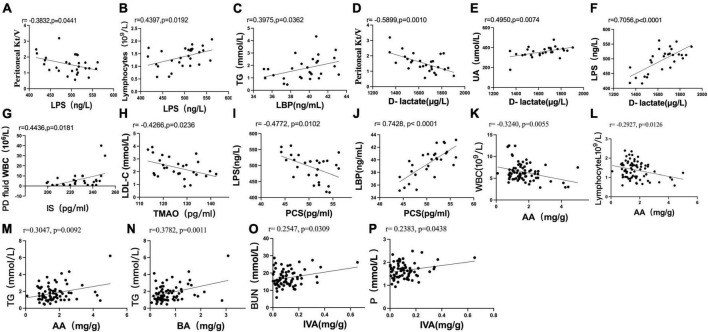
Scatter plots illustrating the statistical correlation between the clinical indicators, serum indicators (LPS, LBP, D-lactate, IS, PCS, TMAO), and fecal levels of SCFAs in the CAPD population. **(A,B)** Between the serum level of LPS and PD Kt/V or lymphocytes. **(C)** Between the serum levels of LBP and TG. **(D–F)** Between the serum level of D-lactate and PD Kt/V, serum levels of UA or LPS. **(G)** Between the serum level of IS and peritoneal dialysis fluid WBC count. **(H)** Between the serum levels of TMAO and LDL-C. **(I,J)** Between the serum level of PCS and LPS or LBP. **(K,L)** Between the fecal level of AA and blood WBC or absolute value of blood lymphocytes. **(M,N)** Between the fecal level of AA or BA and TG. **(O,P)** Relationship between the fecal level of IVA and BUN or serum phosphorus.

Pearson correlation analysis showed that the serum level of LPS was negatively correlated with Kt/V (*r* = −0.3832, *P* = 0.0441) (Figure 6A) and positively correlated with the absolute value of lymphocytes (*r* = 0.4397, *P* = 0.0192) (Figure 6B). The serum level of LBP was positively correlated with TG (*r* = 0.3975, *P* = 0.0362) (Figure 6C). The serum level of D-lactate was negatively correlated with Kt/V (*r* = −0.5899, *P* = 0.0010) (Figure 6D) and positively correlated with UA (*r* = 0.4950, *P* = 0.0074) (Figure 6E). The serum level of D-lactate was positively correlated with the serum level of LPS (*r* = 0.7056, *P* < 0.0001) (Figure 6F).

Pearson analysis showed that the serum level of IS was positively correlated with peritoneal dialysis fluid WBC count (*r* = 0.4436, *P* = 0.0181) (Figure 6G). The serum level of TMAO was negatively correlated with LDL-C (*r* = −0.4266, *P* = 0.0236) (Figure 6H). There was no significant correlation between the serum level of PCS and clinical indexes in PD patients (*P* > 0.05).

The serum level of PCS was negatively correlated with the serum level of LPS (*r* = −0.4772, *P* = 0.0102) (Figure 6I) and positively correlated with the serum level of LBP (*r* = 0.7428, *P* < 0.0001) (Figure 6J). No significant correlation was found between the serum levels of IS and TMAO and the serum levels of LPS, LBP and D-lactate (*P* > 0.05). No significant correlation was found between the fecal levels of SCFAs and the serum levels of LPS, LBP, D-lactate, IS, PCS, and TMAO in PD patients (*P* > 0.05).

Pearson analysis showed that the fecal level of AA was negatively correlated with blood WBCs (*r* = −0.3240, *P* = 0.0055) (Figure 6K) and the absolute value of blood lymphocytes (*r* = −0.2927, *P* = 0.0126) (Figure 6L). The fecal levels of AA (*r* = 0.3047, *P* = 0.0092) (Figure 6M) and BA (*r* = 0.3782, *P* = 0.0011) (Figure 6N) were positively correlated with TG. The fecal level of IVA was positively correlated with BUN (*r* = 0.2547, *P* = 0.0309) (Figure 6O) and serum phosphorus (*r* = 0.2383, *P* = 0.0438) (Figure 6P).

**FIGURE 7:**
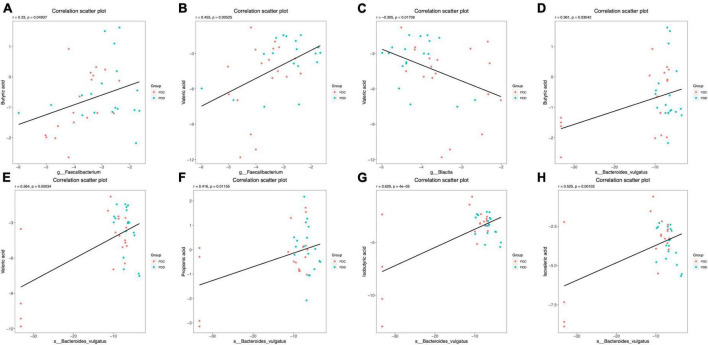
Scatter plots illustrating the statistical correlation between the gut microbiome and the fecal levels of SCFAs of the population in the PDC and PDD groups. **(A,B)** Relationship between the relative abundance of Faecalibacterium and the fecal levels of BA or VA. **(C)** Relationship between the relative abundance of Blautia and the fecal levels of VA. **(D–H)** Relationship between the relative abundance of Bacteroides vulgatus and the fecal levels of BA, VA, PA, IBA, or IVA.

The correlation between the gut microbiome and fecal SCFAs in the PDC and PDD groups was further analyzed. At the genus level, the relative abundance of Faecalibacterium was positively correlated with the fecal levels of BA (*r* = 0.33, *P* = 0.049) (Figure 7A) and VA (*r* = 0.455, *P* = 0.005) (Figure 7B). The relative abundance of Blautia was negatively correlated with the fecal level of VA (*r* = −0.395, *P* = 0.017) (Figure 7C). At the species level, the relative abundance of Bacteroides vulgatus was positively correlated with the fecal levels of BA (*r* = 0.361, *P* = 0.030) (Figure 7D), VA (*r* = 0.564, *P* = 0.000) (Figure 7E), PA (*r* = 0.416, *P* = 0.012) (Figure 7F), IBA (*r* = 0.629, *P* = 4e-05) (Figure 7G), and IVA (*r* = 0.525, *P* = 0.001) (Figure 7H).

## 4 Discussion

In this study, we not only described the differences in gut microbiota and metabolites, serum endotoxin, and serum gut-derived uremic toxin levels between PD patients and healthy people and ESRD patients but also further analyzed PD patients in different dialysis vintages. To the best of our knowledge, this is the first study to comprehensively evaluate the characteristics and differences in intestinal microecology in PD patients during different dialysis vintages by combining indicators such as gut microbiota and metabolites, serum endotoxin, and serum gut-derived uremic toxins. This study is a joint study of the fecal microbiome, fecal and serum targeted metabolomics, and combined clinical data to carry out an association analysis of significant indicators. The research methods involve 16S RNA, GC-MS, and ELISA, aiming to comprehensively analyze the possible mechanisms of the intestinal microecosystem in the long-term treatment of PD and prevention of related complications based on the results of multiomics research.

Previous studies have initially shown that PD treatment affects the diversity and structure of the intestinal flora (11) and the same conclusion was obtained in our study, which found a decrease in diversity, a significant decrease in species evenness, and some differences in the structure of the intestinal flora in CAPD patients compared with healthy subjects and ESRD patients. Under CAPD treatment, the 24 h abdominal retention time of dialysis fluid is as high as 14∼18 h. The prolonged contact of the patient’s intestine with dialysis fluid and the high glycemic and high-pressure abdominal environment are direct factors leading to changes in the intestinal flora. Considering the disease itself, most CAPD patients have completely lost their kidney function or only a small part of their residual kidney function, so they cannot detoxify normally, and many uremic toxins in their bodies, such as urea and uric acid, need to be excreted passively through the intestine, which will affect the intestinal microenvironment, leading to changes in the structure of the intestinal flora. The many oral medications used in the long course of PD treatment can also have an impact on the intestinal flora. In addition, this study further investigated the pattern of intestinal flora changes in CAPD patients at different dialysis vintages for the first time and found that the richness, diversity and evenness of intestinal flora did not change significantly in patients at different dialysis vintages, but there were differences in the composition of intestinal flora. Compared with CAPD patients at dialysis vintages of 3–24 months or more than 60 months, PD patients at dialysis vintages of 24–36 months had significant changes in the structure of intestinal flora. From the analysis of intestinal flora species composition and differential flora, it was found that the dominant intestinal flora of ESRD patients without dialysis treatment were mainly concentrated in Bacteroidia, which differed from CAPD patients with predominantly Firmicutes, and it was known that intestinal flora of CAPD patients were disturbed by both disease factors and dialysis treatment. Although the dominant intestinal flora of both CAPD patients and healthy individuals is concentrated in Firmicutes, anaerobic bacteria such as Ruminococcaceae, Oscillospirales and Faecalibacterium are mainly found in healthy individuals, while the anaerobic bacteria of Blautia and the parthenogenic and aerobic bacteria of Bacilli and Lactobacillales are mainly found in CAPD patients. A decrease in the abundance of anaerobic groups and an increase in the abundance of parthenogenic anaerobic and aerobic groups at the level of Firmicutes is a characteristic feature of intestinal flora disorders in CAPD. It was also shown that CAPD treatment leads to a decrease in the abundance of Bacteroides and Faecalibacterium and an increase in Proteobacteria, Enterobacteriaceae, and Escherichia-Shigella. The abnormal expansion of Enterobacteriaceae was more prominent in CAPD patients with 24 36 months of dialysis vintage. Proteobacteria is also the predominant parthenogenetic anaerobic bacterium, and common Enterobacteriaceae such as Escherichia coli, Salmonella, and Shigella belong to this phylum. The lower abundance of Proteobacteria helps the host to maintain a normal anaerobic environment in the gut, but its abundance is highly susceptible to environmental influences, and abnormal proliferation is associated with a variety of diseases (33). Escherichia coli is one of the main gram-negative bacterial groups that cause enterogenic peritonitis in PD (34), which can enter the abdominal cavity through the damaged intestinal barrier and lead to the occurrence of peritonitis (11). In addition, Escherichia coli and Escherichia Shigella have been proven to be related to intestinal barrier function damage. Cinova (35) et al. found that Escherichia coli CBL2 and Shigella CBD8 have strong adhesion to intestinal epithelial cells and can mediate intestinal mucin secretion and tight junction damage. Pawłowska (36) et al. also found that Escherichia coli can destroy intestinal tight junction proteins and the intestinal epithelial actin cytoskeleton through its own toxic factors and mediate inflammatory responses. Another study (37) found that in the gut of mice with a higher abundance of Proteobacteria, bacteria are more likely to enter the mucosal layer through mucus for bacterial translocation. It can be seen from the above that in terms of the species structure of gut microbiota, PD patients with dialysis vintages of 24 to 36 months had a higher risk of enterogenic peritonitis.

By annotating the microbiota function of all samples in this study, it was found to be closely related to metabolic mechanisms, in which carbohydrate and amino acid metabolism played a major role. Intestinal microorganisms further exert many physiological functions such as metabolism and immunity by helping the host obtain energy from food and regulate host nutrition, among which the fermentation of carbohydrate and amino acid components in food is an important energy source. Compared with healthy people and ESRD patients, the intestinal flora of CAPD patients was weakened in the amino acid metabolism pathway of alanine, aspartic acid and glutamate, but strengthened in the glucose metabolism pathway of starch, sucrose and pyruvate and the nucleotide metabolism pathway of purine and pyrimidine. Pyruvate metabolism was most abundant in CAPD patients with dialysis age ≥24 months and <60 months. Purine and pyrimidine metabolism is widespread in bacteria. Previous studies have found that xanthine deoxy enzyme, which plays an important role in purine metabolism, is highest expressed in liver and intestine (38) and can be secreted by Proteus bacteria such as Escherichia coli (39), and its increased metabolism is considered to be related to the increased abundance of enterobacter in CAPD patients. The microbial pyrimidine metabolic pathway can not only realize the recycling of pyrimidine in the cell, but also provide nitrogen or carbon source for the growth of microorganisms (40, 41), which is enhanced to a certain extent with the metabolic pathway of starch and sucrose, and may be a compensatory energy supply pathway after some amino acid metabolic pathways are weakened in CAPD patients. In recent years, it has been found that Escherichia coli has great application potential in microbial production of pyruvate (42), and natural or engineered Escherichia coli strains have the ability to use glucose and anaerobic conversion into pyruvate. In this study, higher intestinal glucose concentration and enterobacter abundance may be related to the enhancement of pyruvate metabolism of CAPD intestinal flora.

LPS is the main component of the outer membrane of gram-negative bacteria, and LBP is the binding protein of LPS. The expansion of gram-negative bacteria can lead to insufficient clearance and excessive accumulation of LPS, and the combination of the two can stimulate the release of inflammatory factors such as hs-CRP, IL-1, IL-6, and TNF-α through the LPS-LBP-CD14-TLR4 pathway (43). Inflammatory factors not only cause local inflammation in the intestine but also enter the blood or other organs and tissues with the circulation, thus triggering microinflammatory responses in other parts of the body and even the whole body (44, 45). In this study, CAPD patients with dialysis vintage of 24∼36 months had a higher abundance of intestinal gram-negative bacteria and higher serum LPS concentrations than patients with other dialysis vintages, while serum LBP was at a lower level, especially compared to patients with more than 60 months of dialysis vintage, and the increase in serum concentrations of both was not parallel. It has been suggested that the toxic effect of LPS is only dependent on the sensitizing effect of LBP within a certain range and that the sensitizing effect of LPS is reduced or even disappears when LBP increases to a certain concentration. High concentrations of LPS can increase cytokines and inflammatory mediators through TLR4 pathway activation of target cells independent of the sensitizing effect of LBP. At the same time, a high concentration of LBP also has a protective effect, which not only has a direct neutralizing effect of LPS but can also negatively regulate the activation of mononuclear macrophages to reduce the release of inflammatory factors and can eliminate some gram-negative bacteria by enhancing the binding of macrophages and Escherichia coli and its phagocytosis. In correlation analysis, the serum level of LPS in CAPD patients was positively correlated with absolute blood lymphocyte values, elevated lymphocytes were associated with the infection status of the organism, and LPS and its proinflammatory factors may be the source of infection (46). The serum level of LPS was negatively correlated with PD Kt/V, suggesting that accumulation of LPS may decrease the efficacy of PD, which is considered to be related to LPS exacerbating peritoneal mesothelial cell injury and promoting peritoneal fibrosis (47, 48). The serum level of LBP was positively correlated with TG, and some studies have shown that a higher serum level of LBP is associated with the risks of obesity, metabolic syndrome, type 2 diabetes and atherosclerosis (49, 50), which may be related to the innate immune response triggered by LBP leads to metabolic disturbances (51).

D-Lactate is a unique fermentation metabolite of a variety of bacteria in the gut. Normally, the serum level of D-Lactate is very low. When a large number of bacteria in the digestive tract are digested, the intestinal barrier is damaged, and intestinal permeability is increased. D-Lactate enters the blood circulation through the intestinal mucosa, resulting in elevated serum levels. Monitoring the level of serum D-Lactate can reflect the changes of intestinal mucosal damage and permeability in time (52). In this study, serum D-Lactate in CAPD and ESRD patients was significantly higher than that in healthy people, suggesting different degrees of intestinal barrier damage and increased permeability in both patients, while serum D-Lactate level in CAPD patients was significantly lower than that in ESRD patients, indicating that CAPD treatment did not cause further damage to the intestinal barrier. And may have played a repairing role. Correlation analysis showed that serum D-Lactate level was positively correlated with LPS level and abdominal permeation fluid WBC, and negatively correlated with PD-KT/V. Studies have shown that LPS can directly destroy the intestinal barrier (36, 53), increase intestinal permeability and bacterial translocation (37, 54), and after intestinal barrier damage, the intestinal barrier can be reduced. Abnormal bacteria, LPS and other metabolic toxins enter the abdominal cavity, which not only increase the risk of abdominal infection but also destroy the peritoneal structure and thus affect the efficiency of peritoneal dialysis. It was also found that serum D-Lactate level was positively correlated with UA, indicating that high uric acid may also be the cause of intestinal barrier damage.

Chronic kidney disease is characterized by decreased clearance of metabolic wastes, namely, uremic toxin, and increased serum accumulation. Gut-derived metabolites are also an important part of uremic toxins. The most studied gut-derived uremic toxins were IS, PCS and TMAO. Phenols are the fermentation products of tyrosine and phenylalanine produced by intestinal bacteria, and indole is produced by intestinal bacteria through the proteolysis of tryptophan and then further forms IS and PCS by hepatic sulfation. When renal function is normal, they are mainly cleared by the kidneys through renal tubular secretion (55), and renal function is damaged, which leads to the accumulation of IS and PCS, resulting in toxic effects. IS accumulation has been suggested to be associated with increased fibrosis and oxidative stress (13). PCS and its precursor (unconjugated p-cresol, uPC) have been found to be associated with CKD insulin resistance (16), dyslipidemia, atherosclerosis, and increased cardiovascular risk (15, 56). TMAO is an intestinal metabolite formed under the action of intestinal microorganisms by eating foods rich in carnitine, phosphatidylcholine, betaine, and L-carnitine, of which more than 95% are excreted by glomerular filtration (57). It accumulates in the body when kidney function is impaired. TMAO is an amine small molecule compound that can enter the blood. Studies have confirmed that the level of circulating TMAO increases with decreasing renal function, which is related to the prognosis of CKD (58, 59), microvascular and macrovascular complications (60) and adverse cardiovascular events (17). In this study, the serum level of IS in ESRD patients was significantly higher than that in healthy subjects and CAPD patients, indicating that the CAPD treatment modality has a clearing effect on IS and is more pronounced in patients with a dialysis vintage of 12 to 24 months. The serum levels of IS in patients with a dialysis vintage of 3 to 12 months or more than 60 months were still higher than those in healthy subjects. Serum levels of PCS and TMAO in CAPD patients were significantly higher than those in healthy people and ESRD patients. The accumulation of PCS was more significant in patients at the early stage of dialysis (3∼12 months) and at the late stage (over 60 months), while the accumulation of TMAO was higher in patients at the middle stage of dialysis (12 ∼ 36 months). The above results suggested that IS, PCS, and TMAO may still play a toxic role in the long course of CAPD. IS, PCS, and TMAO in the human body are mainly derived from the enzymatic digestion of exogenous food by the flora, and their biosynthesis and circulating levels are related to various factors, such as intestinal flora, dietary structure and liver metabolism. It has been found that ESRD patients tend to relax the restriction of a high-protein diet after receiving PD treatment in clinical practice, which may be one of the factors leading to higher serum concentrations of IS and PCS in the early stage of dialysis and a decrease after routine PD treatment and standardized diet management. High concentrations in the late stage of dialysis may be associated with declining residual kidney function, which has been shown to account for 80% of total daily IS and PCS clearance through the residual kidney, while dialysis accounts for only approximately 20% (61), suggesting that residual kidney function plays a major role in the clearance of IS and PCS. Serum levels of TMAO were lower in CAPD patients with a dialysis vintage of more than 60 months, which was consistent with the better intestinal flora structure, lower serum level of LPS and milder degree of intestinal barrier damage, suggesting a closer relationship between TMAO and the intestinal environment. In the correlation analysis, the serum level of IS in CAPD patients was positively correlated with the leukocyte count in the abdominal dialysis fluid, tentatively indicating that IS may be a risk factor for abdominal inflammation. In addition, the serum level of TMAO in CAPD patients was negatively correlated with LDL-C,which suggests that TMAO may be an independent factor of cardiovascular risk in CAPD patients distinct from LDL-C. This study also found the association of serum levels of PCS with LPS and LBP in CAPD patients, and the interactions and impacts on disease still need to be further explored and studied.

Short-chain fatty acids are mainly derived from dietary fiber, complex carbohydrates (resistant starch, non-starch polysaccharides, indigestible oligosaccharides, and sugar alcohols) and undigested proteins or peptides in the gut (62–64). As a major metabolite of intestinal flora, SCFAs can not only provide energy for intestinal epithelial cells but also relieve intestinal inflammation by reducing the production of proinflammatory factors (64, 65). SCFAs play an important role in maintaining intestinal function and integrity. In addition, SCFAs can enter the blood circulation as signaling molecules and play biological roles in peripheral tissues and the systemic circulatory system. In recent years, several studies have shown that SCFAs can protect the kidney by inhibiting inflammation, reducing oxidative stress, decreasing apoptosis, promoting autophagy, regulating blood pressure, and regulating glucolipid metabolism (21), especially BA, which has the most prominent protective effect on the kidney (66). The content of SCFAs in feces is correlated with the structure of the intestinal flora. Previous studies have shown that CAPD patients have a lower abundance of Bacteroides and Faecalibacterium. Bacteroides is the dominant bacteria in the human gut (67), which can not only directly inhibit the adhesion and growth of other harmful intestinal flora through its own colonization but also ferments difficult to digest macromolecules of carbohydrates, providing energy for the host and converting them into SCFAs. Faecalibacterium is also an important butyric acid-producing bacterium (63), whose anti-inflammatory effect has been confirmed *in vivo* and *in vitro* (68) and is believed to be achieved through the production of butyrate, which can maintain the intestinal barrier and reduce inflammation (69). In the present study, fecal levels of AA, BA, VA, and CA were reduced in CAPD patients compared to healthy subjects, with a significant decrease in BA content. The decreased fecal levels of various SCFAs, especially BA, were considered to be related to the decreased relative abundance of Bacteroides and Faecalibacterium. Patients with a dialysis vintage of more than 60 months had a higher abundance of Faecalibacterium and Bacteroides when their fecal levels of AA, BA, PA, and VA were also higher. Since there was a significant difference in the microflora of CAPD patients with dialysis vintages of 24∼36 months and those over 60 months, we focused on analyzing the correlation between the differential bacteria and fecal levels of SCFAs of these subjects. The results showed that a variety of SCFAs were positively correlated with Faecalibacterium and Bacteroides vulgatus. Fecal levels of IBA and IVA were higher in CAPD and ESRD patients than in healthy subjects and were higher in CAPD patients with 24∼36 months of dialysis vintage. Correlation studies preliminarily found that the fecal level of IVA in CAPD patients was positively correlated with serum BUN and serum phosphorus, suggesting that fecal IVA may have harmful effects in CAPD patients. Fecal levels of AA were negatively correlated with serum WBCs and lymphocyte levels, suggesting that fecal AA may have a role in reducing inflammation and regulating immunity in CAPD patients. Correlation analysis also found that fecal levels of AA and BA were associated with elevated triglycerides in CAPD patients. Previous studies have been divided in understanding the relationship between SCFAs and lipid metabolism. Some studies believed that SCFAs played a beneficial role in regulating lipid metabolism disorders (70, 71), but some studies also found that higher SCFAs were associated with central obesity and dyslipidemia (72).

This study has several limitations. First, due to the large number of male patients among the disease sources available for screening, gender imbalance occurred during inclusion. Although we have tried to control various influencing factors such as age, region, inclusion period, nutrition and biochemical indicators, the interference of gender cannot be completely excluded. Second, CAPD patients take a variety of oral drugs at the same time to control the primary disease and complications. Although the influence of major drugs such as diabetes and hypoglycemic drugs, antibiotics and probiotics has been excluded, it cannot be ruled out that some patients may take other drugs that affect the flora, such as lipid-lowering drugs. In the future, we will improve the control of the above variables, carry out randomized controlled trials on the intervention of probiotics, metabolites or high-fiber foods, and increase the inclusion of inflammatory status and complications risk assessment indicators such as frequency of peritonitis, vascular CT and cardiac color Doppler ultrasound, so as to further evaluate the role of intestinal microecosystem in PD treatment.

## 5 Conclusion

In summary, we were pleased to find that PD patients on dialysis for more than 60 months showed a good trend in terms of flora composition, serum LPS, serum D-lactic acid, and fecal SCFAs, which suggested that the intestinal microecology of PD patients had a certain ability of self-regulation and remodeling under the management of standardized system. We speculate that the comprehensive state of CKD patients in the standard and continuous PD routine treatment tends to be stable and showing a positive trend, which was conducive to the long-term application of PD and further development. In contrast, it is necessary to strengthen the monitoring of the intestinal status and the occurrence of related complications in PD patients with 24∼36 months of dialysis vintage. Incorporating intestinal microecosystem monitoring into the long-term management of CAPD patients is a new strategy, and it is initially considered that patients with dialysis age ≥24 months and <60 months are the key clinical management objects. In addition, this study provides much support for the application prospects and application timing of probiotics and SCFAs in PD patients, and we believe that Bacteroides, Faecalibacterium prausnitzii and butyric acid have great application potential.

## Data availability statement

The datasets presented in this study can be found in online repositories. The names of the repository/repositories and accession number(s) can be found below: The BioProject database (https://www.ncbi.nlm.nih.gov/bioproject/?term=PRJNA863067) with accession number PRJNA863067.

## Ethics statement

The studies involving humans were approved by the Institutional Review Board of First Teaching Hospital of Tianjin University of Traditional Chinese Medicine. The studies were conducted in accordance with the local legislation and institutional requirements. The participants provided their written informed consent to participate in this study.

## Author contributions

JL: Writing – original draft, Writing – review and editing. HX: Funding acquisition, Writing – review and editing. WL: Methodology, Writing – review and editing. HaY: Formal analysis, Writing – review and editing. BY: Methodology, Writing – review and editing. CJ: Writing – review and editing. JZ: Writing – review and editing. RW: Writing – review and editing. FD: Writing – review and editing. MP: Writing – review and editing. HoY: Writing – review and editing.
