# Supplementary material for: Specific gut microbiome and metabolome changes in patients with continuous ambulatory peritoneal dialysis and comparison between patients with different dialysis vintages
Source: Front Med (Lausanne). 2024 Jan 5;10:1302352. doi: 10.3389/fmed.2023.1302352 (PMC10797064; doi:10.3389/fmed.2023.1302352)
Supplement: Supplementary file 1 [file Table_1.docx]

**Supplementary Appendix**

**Table S1** Clinical laboratory indicators of the CAPD population.

| Variables | PDA | PDB | PDC | PDD | p value |  |
| --- | --- | --- | --- | --- | --- | --- |
| RBC (10^9^/L) | 3.94±0.48 | 3.88±0.44 | 4.03±0.70 | 3.68±0.38 | 0.207 |  |
| WBC (10^12^/L) | 6.47±0.48 | 6.50±1.74 | 6.43±1.50 | 6.15±2.54 | 0.952 |  |
| PLT (10^9^/L) | 205±80 | 218±50 | 197±64 | 211±64 | 0.788 |  |
| Neutrophils (10^9^/L) | 4.35±2.01 | 4.48±1.42 | 4.13±0.99 | 4.4±2.09 | 0.935 |  |
| Neutrophils (%) | 66.2±8.3 | 68.6±8.6 | 64.4±6.9 | 69.9±6.6 | 0.143 |  |
| Lymphocytes (10^9^/L) | 1.49±0.45 | 1.34±0.48 | 1.54±0.38 | 1.17±0.39 | 0.049 |  |
| Lymphocytes (%) | 23.9±6.8 | 21.1±7.2 | 24.2±5.1 | 20.4±6.1 | 0.168 |  |
| Monocytes (10^9^/L) | 0.37±0.1 | 0.41±0.14 | 0.39±0.11 | 0.34±0.13 | 0.358 |  |
| Monocytes (%) | 5.9±1.5 | 6.2±1 | 6.1±1.3 | 5.8±1.7 | 0.804 |  |
| HGB (g/L) | 119±12 | 119±10 | 124±22 | 116±9 | 0.429 |  |
| TG (mmol/L) | 1.76±0.88 | 1.65±0.93 | 1.72±0.91 | 2.07±1.27 | 0.613 |  |
| TC (mmol/L) | 4.34±1 | 4.08±0.98 | 3.78±0.69 | 4.16±0.91 | 0.317 |  |
| LDL-C (mmol/L) | 2.37±0.86 | 2.18±0.78 | 1.98±0.5 | 2.12±0.77 | 0.457 |  |
| HDL-C (mmol/L) | 1.08±0.36 | 1±0.2 | 0.92±0.27 | 0.97±0.21 | 0.338 |  |
| CR (umol/L) | 833.12±232.33 | 966.26±234.72 | 980.78±375.83 | 928.38±297.63 | 0.427 |  |
| BUN (mmol/L) | 16.82±5.12 | 18.23±4.59 | 18.65±4.38 | 15.17±4.48 | 0.114 |  |
| UA (umol/L) | 380.93±50.37 | 378.6±47.88 | 374.2±39.41 | 356.8±62.3 | 0.479 |  |
| eGFR (ml/min/m^2^) | 5.55±2.18 | 5.09±1.81 | 4.88±2.36 | 5.64±5.7 | 0.892 |  |
| ALB (g/L) | 36.1±5.8 | 37.4±3.9 | 35.8±3.8 | 37±3.6 | 0.677 |  |
| 𝛾-GGT (U/L) | 15±10.4 | 18.6±9.6 | 22.7±13.4 | 24±21.1 | 0.232 |  |
| serum K (mmol/L) | 4.46±0.67 | 4.08±0.6 | 4.16±0.6 | 4.1±0.63 | 0.227 |  |
| serum Ca (mmol/L) | 2.13±0.21 | 2.17±0.09 | 2.12±0.17 | 2.13±0.26 | 0.838 |  |
| serum P (mmol/L) | 1.72±0.35 | 1.52±0.28 | 1.74±0.23 | 1.62±0.41 | 0.182 |  |
| iPTH (pg/ml) | 386.89±224.15 | 528.64±193.51 | 514.66±281.94 | 686.16±404.82 | 0.026 |  |
| residual renal Kt/V | 0.774±0.585 | 0.547±0.478 | 0.456±0.675 | 0.419±0.756 | 0.338 |  |
| Peritoneal Kt/V | 1.305±0.468 | 1.395±0.392 | 1.569±0.28 | 1.699±0.639 | 0.059 |  |
| Peritoneal CCr | 38.574±33.175 | 27.674±22.640 | 26.964±36.099 | 18.430±33.423 | 0.310 |  |
| residual renal CCr | 36.804±14.812 | 42.388±10.085 | 39.863±12.804 | 36.5±9.617 | 0.414 |  |
| nPCR | 1.024±0.534 | 1.163±1.019 | 1.037±0.513 | 1.477±1.704 | 0.549 |  |
| PD fluid WBC (10^6^/L) | 11±13 | 5±5 | 4±3 | 6±12 | 0.115 |  |
| PD fluid GLU (mmol/L) | 31.03±11 | 31.76±13.94 | 33.43±9.71 | 34.52±12.64 | 0.813 |  |

Values are expressed as absolute number or mean ± SD.
